# Biogenic silver nanoparticles: Synthesis, applications and challenges in food sector with special emphasis on aquaculture

**DOI:** 10.1016/j.fochx.2023.101051

**Published:** 2023-12-07

**Authors:** Saba Khursheed, Joydeep Dutta, Ishtiyaq Ahmad, Mohd Ashraf Rather, Irfan Ashraf Badroo, Tashooq Ahmad Bhat, Irfan Ahmad, Adnan Amin, Azra Shah, Tahiya Qadri, Huraiya Habib

**Affiliations:** a Department of Zoology, School of Bioengineering & Biosciences, Lovely Professional University, Phagwara, Punjab 144411, India; b Division of Fish Genetics and Biotechnology, Faculty of Fisheries Ganderbal, Sher-e- Kashmir University of Agricultural Science and Technology, Kashmir 190006, India; c Government Degree College Women Sopore, Kashmir, Jammu and Kashmir 193201, India; d Division of Food Science and Technology, Sher-e-Kashmir University of Agricultural Sciences and Technology-Kashmir, Jammu and Kashmir 190025, India; e Division of Aquatic Environmental Management, Faculty of Fisheries, Rangil, Ganderbal, SKUAST-Kashmir, 190006, India; f Department of Food Technology and Nutrition, Lovely Professional University, Phagwara, Punjab, India

**Keywords:** Silver nanoparticles (AgNPs), Aquaculture, Water quality, Fish disease

## Abstract

Aquaculture, a rapidly expanding global food sector faces challenges like pathogenic infections, water quality management and sustainability. Silver nanoparticles (AgNPs) have emerged as promising tools in aquaculture due to their antimicrobial, antiviral and antifungal properties. AgNPs offer alternatives to traditional antimicrobial agents. Their small size and unique physicochemical properties enhance antimicrobial activity, effectively inhibiting pathogen growth and reducing disease incidence in aquatic organisms. Additionally, AgNPs can improve water quality by catalyzing the removal of pollutants, heavy metals and nutrients, reducing environmental impacts. Despite their potential benefits, several challenges and knowledge gaps exist in the utilization of AgNPs in aquaculture. Addressing challenges related to regulation, sustainability and environmental impact will be crucial for realizing their full potential in the industry. Therefore, the present review aims to provide insight into the role of AgNPs, its challenges in aquaculture and also highlights key areas for future research.

## Highlights

• Silver nanoparticles, synthesized from natural sources, offer an eco-friendly alternative to conventional chemical methods.
• Silver nanoparticles exhibit strong antimicrobial properties, pivotal for safeguarding fish health.
• Silver nanoparticles in food packaging materials can extend shelf life, reducing food waste and enhancing seafood freshness during transport and storage.

## Introduction

Aquaculture is regarded as one of the most important food production systems in terms of both economic effect and food security and its continued expansion is a critical component of the strategy to ensure global nutritional safety (Fajardo et al., 2022). Food security is a major global issue with millions of people globally suffering from malnutrition and hunger. Therefore, it requires an immediate action if we are to construct a better and more sustainable future. Aquaculture technology can contribute to addressing this issue by increasing the availability and accessibility of fish and other aquatic foods, providing a source of protein-rich food, improving nutrition, promoting sustainability, creating income and improving food safety. Different forms of nanotechnology-based systems are being used to increase production, efficiency and sustainability. Nanotechnology and nanoscience are extremely promising fields and quickly growing topics in scientific and technological innovation. Nanotechnology exhibits various multidisciplinary practises in both agriculture and aquaculture. A nanoparticle (NP) is commonly defined as a structure with a size between 0.1 and 100 nm (Nasr-Eldahan et al., 2021). Numerous industries are discovering the potential benefits of nanotechnology and products based on nanotechnologies or involving nanomaterials that have already been created in the fields of electronic components, consumer goods and pharmaceuticals. A wide range of numerous applications in agriculture and food are also emerging, including packaging technologies, nano sensors for disease detection or conditions of storage, nano compositions of agricultural chemicals and nano transportation of foodstuffs. Nanotechnology, which has grown into a multibillion-dollar business worldwide, produces various nanostructures such as metal-oxide nanoparticles, tiny quantum dots, carbon nanotubes, fullerenes and so on (Aitken et al., 2006, Akcan et al., 2020). Due to their small size and high surface-to-charge ratio, they are incredibly reactive with increasing characteristics, attracting the attention of their usage in a variety of sectors.

On the other hand, same properties have also made nanoparticles a common pollutant by increasing their toxicity in the environment (Nowack, 2008). They can leak and penetrate water bodies during production, delivery and use causing harm to ecosystems and other living things even humans (Chen & Huang, 2017). They are transported through to terrestrial and aquatic environments by a variety of routes, where they are encountered by living animals. As a result, it encouraged researchers to adopt the preparation of nanoparticles using plant-based extracts in the synthesis of nanoparticles using sustainable techniques, as the plants are easily accessible, readily available and considerably less hazardous to work with, as well as serve as the source for multiple metabolites.

A considerable amount of research is currently being conducted on the synthesis of nanoscale metals employing physical, chemical and environmentally sound synthesis procedures (Ying et al., 2022). Because of issues with high energy consumption, toxic and harmful substances get released. Green synthesis methods are gradually replacing physical and chemical procedures due to the use of advanced equipment and synthesis conditions (Ahmed & Ikram, 2015). At the moment, microorganisms (fungi, bacteria and algae) are mostly used in green synthesis or extracts from leaves, roots, peelings (Ehrampoush, Miria, Salmani, & Mahvi, 2015), flowers (Sone, Diallo, Fuku, Gurib-Fakim, & Maaza, 2020), fruits (Kumar et al., 2017) and seeds (Gao, Li, Pan, & Si, 2016) of various plants. Polyphenols and proteins found in green materials can function as reducing agents in place of chemical reagents to change metal ions into lower valence states (Can, 2020). Compared to chemical and physical techniques, green synthesis offers a number of benefits, such as being non-toxic, pollutant-free, environmentally friendly, inexpensive and more ecologically sound.

A variety of processes are used in the synthesis of silver nanoparticles involves a number of procedures such as the thermal breakdown of organic solvents, photo reduction in reverse micelles and chemical reduction of silver ions. All of these solutions, however, are costly and involve the use of harmful chemicals that endanger the natural environment. Because of the negative impacts of chemical synthesis, biological techniques using enzymes have been developed (Talekar, Joshi, Chougle, Nakhe, & Bhojwani, 2016), microorganisms (Prasad, Pandey, & Barman, 2016), and plants (Song & Kim, 2009) were used for the synthesis of silver nanoparticles providing eco-friendly, rapid, and low-cost attributes. Due to its eco-friendly, inexpensive, and secure nature for usage in human medicinal applications, plant-based nanoparticle production is more acceptable among these biological techniques.

The aquaculture industry has grown recently to meet the growing need for foodstuffs from the growing global population (Arechavala‐Lopez, Cabrera‐Álvarez, Maia, & Saraiva, 2022). The business has attracted a lot of interest despite being relatively new in comparison to farming and fishing. Fish and shellfish diseases caused by pathogenic bacteria and viruses pose a serious threat to the sector and have resulted in large scale financial losses (De Silva et al., 2021). Together with infections, poor water quality is a concern that has been linked to the development of diseases in aquaculture products. Over the years, silver nanoparticles have drawn attention as one of the most successful nanoparticles, which has been utilized in a range of fields of study, with the greatest success as antibacterial agents (Zhao et al., 2023). Although there are in vitro outcomes, however, there is lack of vivo results for the use of AgNPs in aquaculture and livestock as of yet (De Silva et al., 2021). The use of AgNPs in aquaculture is essential to control diseases that threaten the industry, causing product loss and resulting in financial losses (Elayaraja et al., 2021). The use of AgNPs in aquaculture is essential for reducing problems caused by aquatic illnesses and hence increasing economic value (Shah & Mraz, 2020). Aquatic life in ponds can be produced more of and survive at higher rates when AgNPs are used in aquaculture. Therefore, the purpose of this review is to critically evaluate the existing knowledge on the use of synthetic silver nanoparticles derived from plants and their applications in aquaculture, as well as the challenges associated with silver nanoparticle toxicity and to highlight key areas for future research.

## General overview of silver nanoparticles

The most researched nano-antibacterial agent is silver nanoparticles. In contrary to antibiotics, which typically operate by a single process (Adeniji, Nontongana, Okoh, & Okoh, 2022). Silver nanoparticles combat pathogenic bacteria via numerous pathways, allowing them to overcome resistance to antibiotics. Silver nanoparticles antibacterial mechanism has yet to be fully discovered. Researchers have proposed numerous tools that may be associated with changes in the structure and morphology of bacterial cells caused by nanoparticle size with respect to their high volume compared to surface area and shape, the small size of the nanoparticles allows for greater contact with the cells of bacteria and easier absorption. The antibacterial effect of silver nanoparticles is greatly influenced by these physiochemical parameters. Silver nanoparticles are considered to bind to the cell surface via electromagnetic charges, in which the positively charged charge on the exterior of the nanoparticle positively connects to the negative electrical charge of the cell membrane, enhancing nanoparticle membrane adherence. Nanoparticles allow the discharge of Ag ^+^ ions that attach with microorganism cell wall protein molecules, causing alterations to cell structure of membranes, including flexibility, and ultimately cell death. They can also bind to mitochondrial and nucleonic acids, generating damage to cells and interfering with the division of cells. As an antifungal medication, silver nanoparticles demonstrated high inhibitory properties against Candida species, comparable to the commercialised antifungal Amphotericin B (sanjemban). There has been little research, particularly on the medicinal use of antifungal and antivirus silver nanoparticles in aquaculture.

## Synthesis of silver nanoparticles

**Fig. 1:**
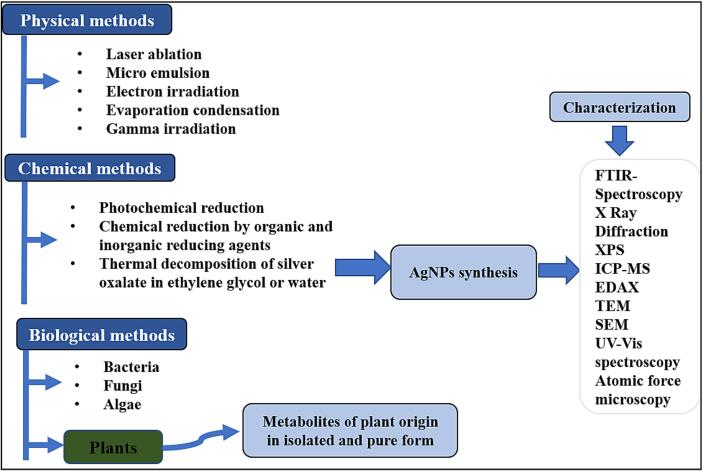
Commonly used methods (chemical, physical, and biological) for synthesis of silver nanoparticles and the techniques used for the characterization of synthesized nanoparticles.

Silver nanoparticles have typically been produced through physical and chemical techniques, but recent research has focused on the crucial involvement of biological systems in this process (Fig. 1). Plant-derived silver nanoparticles (AgNPs) are among the simplest to formulate. A silver metal ion solution and a reducing biological agent are required for the green production of silver nanoparticles. Tolaymat et al. (2010) describe the easiest and least expensive method of producing AgNPs as reducing and stabilising Ag ions with a combination of biomolecules such as polysaccharides, vitamins, amino acids, proteins, phenolics, saponins, alkaloids, and/or terpenes. Almost all plants have the potential to be employed in AgNPs synthesis. Furthermore, plant extracts are found to be more suitable candidates for bio-inspired synthesis of AgNPs than other biological entities (microorganisms and fungi) because they do not necessitate the use of toxic reducing and capping agents, radiation, microbial or fungal strains, or other nanoparticle production processes.

**Fig. 2:**
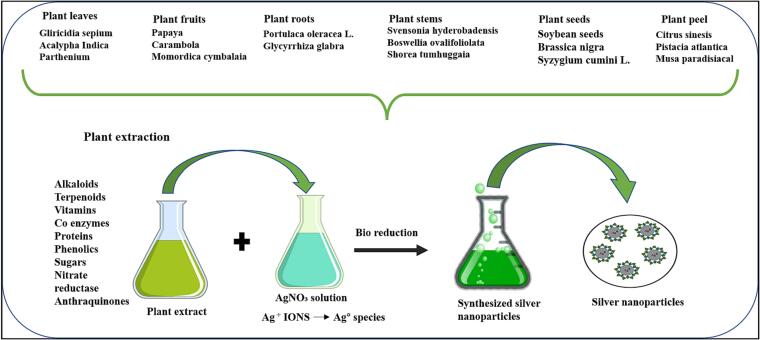
Steps involved in the synthesis of silver nanoparticles.

They also lower the risk of contamination and infection throughout the synthesis and application processes. Because of the slower kinetics, it also allows for better regulation and oversight of crystal stabilisation growth. Plant extracts are prioritised economically due to their abundant availability. Furthermore, extract preparation is a low-cost and simple procedure. Fig. 2 depicts the various steps of plant-mediated nanoparticle formation. Table 1 shows silver nanoparticles made from plant leaves, fruits, flowers, roots, stems, and seeds.

**Table 1:** Use of different part of the plants to synthesis silver nanoparticles.

	Scientific Name of the Plant	Size	References
Plant leaf	*Gliricidia sepium*	27 nm	(Raut Rajesh et al., 2009)
	*Parthenium*	50 nm	(Parashar et al., 2009)
	*Acalypha Indica*	20–30 nm	(Krishnaraj et al., 2010)
	*Coriandrum sativum*	26 nm	(Sathyavathi et al., 2010)
	*Euphorbia hirta*	40–50 nm	(Elumalai et al., 2010)
	*Lippia citriodora*	15–30 nm	(Cruz et al., 2010)
	*Euphorbia hirta*	40–50 nm	(Elumalai et al., 2010)
	*Desmodium triflorum*	10 nm	(Ahmad et al., 2011)
	*Phyllanthus amarus*	32–53 nm	(Annamalai et al., 2011)
	*Hevea brasiliensis*	401–434 nm	(Mondal et al., 2011)
	*Cephalandra indica*	40–90 nm	(Celin Hemalatha & Renitta, 2011)
	*Catharanthus roseus*	48–67 nm	(Bharath et al., 2012)
	*Ficus carica*	10–20 nm	(Singh & Bhakat, 2012)
	*Citrus limon*	>100 nm	(Vankar & Shukla, 2012)
	*Catharanthus roseus*	35–55 nm	(Kulkarni et al., 2012)
	*Elaeagnus latifolia*	30–50 nm	(Phanjom et al., 2012)
	*Nerium oleander*	48–67 nm	(Sugany et al., 2012)
	*Dalbergia sissoo*	5–55 nm	(Singh et al., 2012)
	*Cassia auriculata*	420–435 nm	(Parveen et al., 2012)
	*Chromolaena odorata*	40–70 nm	(Geetha et al., 2012)
	*Coleus aromaticus*	44 nm	(Vanaja & Annadurai, 2013)
	*Paederia foetida*	24 nm	(Lavanaya et al., 2013)
	*Elaeagnus indica*	30 nm	(Natarajan et al., 2013)
	*Arbutus unedo*	9–15 nm	(Srinivas et al., 2013)
	*Bixa orellana*	35–65 nm	(Thilagam et al., 2013)
	*Ocimum bacillicum*	58–89 nm	(Sivarangani, 2013)
	*Odina wodier*	5–30 nm	(Kumar et al., 2013)
	*Ceratonia siliqua* L.	5–40 nm	(Awwad et al., 2013)
	*Juglans regia* L.	10–50 nm	(Korbekandi et al., 2013)
	*Sonchus asper*	2–100 nm	(Verma et al., 2013)
	*Phyllanthus reticulatus*	11–30 nm	(Kudle et al., 2013)
	*Azhadirachta indica*	21.07 nm	(Lalitha et al., 2013)

Fruit of plants	*Carica papaya*	10–50 nm	(Jain et al., 2009)
	*Musa paradisiaca*	300 nm	(Bankar et al., 2010)
	*Vitis vinifera*	10–50 nm	(Korbekandi et al., 2013)
	*V. Vinifera*	37–44 nm	(Gnanajobitha et al., 2013)
	*Piper longum*	46 nm	(Reddy et al., 2014)
	*Lycopersicon esculentum*	10–40 nm	(Maiti et al., 2014)
	Carambola	10 to 40 nm	(Gavade et al., 2015)
	*Momordica cymbalaria*	15.5 nm	(Swamy et al., 2015)

Flower extracts	*Boswellia serrate*	60–84 nm	(Kudle et al., 2000)
	*Mirabilis jalapa*	60–70 nm	(Vankar & Bajpai, 2010)
	*Cassia auriculata*	10–40 nm	(Velavan et al., 2012)
	*Carthamus tinctorius* L.	Between 40 and 200 nm	(Nagaraj et al., 2012)
	*Ipomoea indica*	10–50 nm	(Pavani et al., 2013)
	*Plumeria alba*	10–50 nm	(Nagaraj et al., 2013)
	*Millingtonia hortensis*	10–40 nm	(Gnanajobitha et al., 2013)
	*Gnidia glauca*	5–20 nm	(Patil et al., 2013)

Root of plant	*Glycyrrhiza glabra*	20–30 nm	(Dinesh et al., 2012)
	*Portulaca oleracea* L.	146 nm	(Asghari et al., 2014)

Stem of plant	*Boswellia ovalifoliolata*	30–40 nm	(Ankanna et al., 2010)
	*Shorea tumbuggaia*	40 nm	(Savithramma et al., 2011)
	*Svensonia hyderobadensis*	300 nm	(Rao & Savithramma, 2012)
	*Portulaca oleracea* L.	146 nm	(Gholamreza et al., 2014)

Seed of plant	*Syzygium cumini* L.	450 nm	(Banerjee & Narendhirakannan, 2011)
	*Glycine* max	25–50 nm	(Prasad & Venkateswarlu, 2014)
	*Brassica nigra*	41 nm	(Pandit, 2015)

Peel of plant	*Butyrospermum paradoxum*	421 nm	(Ajayi et al., 2015)
	*Pistacia atlantica*	10–50 nm	(Sadeghi et al., 2015)
	*Psoralea corylifolia*	100–110 nm	(Sunita et al., 2014)
	*Argyreia nervosa*	20–50 nm	(Thombre et al., 2014)
	*Eucalyptus hybrid*	50–150 nm	(Dubey et al., 2009)
	*Citrus sinesis*	10–35 nm	(Kaviya et al., 2011)
	*Musa paradisiacal*	20 nm	(Bankar et al., 2010)

## Characterization of silver nanoparticles

Characterization of silver nanoparticles is an essential step in determining the functional effect of synthesized particles. Numerous studies have demonstrated that the biological activity of silver nanoparticles is modulated by their morphology, structure, shape, size, charge and coating/capping, chemical structure, redox capacity, nanoparticle separation, ion release, and degree of agglomeration (Siddiqi, Husen, & Rao, 2018). These parameters can be determined using a variety of analytical methods including X-ray diffraction (XRD), Ultra-violet visible spectroscopy (UV–vis), Scanning electron microscopy (SEM), Fourier Transform Infrared (FTIR) spectroscopy, Transmission electron microscope (TEM), X-ray photoelectron spectroscopy (XPS), Zeta potential and Dynamic light scattering (DLS). Silver nanoparticles of modest size have the ability to penetrate numerous biological cell membranes, such as bacterial cell walls, thereby expanding their contact area and ability to easily enter the cell.

## Use of silver nanoparticles in aquaculture

Nanotechnology holds the potential to revolutionise the fisheries and aquaculture industries by providing new tools such as rapid disease diagnosis and improved fish absorption of pharmaceuticals like hormones, vaccinations and nutrition. In 2014 The Food and Agricultural Organization (FAO) release a report indicating a recent surge in aquaculture. In contrast to farming and fishing, this was relatively modern activity. The practise of aquaculture has continuously changed to address issues like infections brought on by bacteria and viruses (Lieke et al., 2020). Moreover, excessive use of water resources and effluent contamination caused by industry waste is a major issue. To overcome the obstacles preventing their development, the aquaculture industry must use innovative technologies.

### Effect of silver nanoparticles on fish growth

The effect of silver nanoparticles on fish growth can vary based on various factors, including nanoparticle concentrations, fish size and the duration of exposure. Studies have shown that exposure to silver nanoparticles can have both positive and negative impacts on fish growth (Table 2). On the one hand, silver nanoparticles have been shown to improve the growth and survival of certain fish species by improving their immune function and enhancing their ability to resist disease (Ochoa-Meza et al., 2019). On the other hand, exposure to high concentrations of silver nanoparticles can also have toxic effects on fish, including reduced growth rates, decreased feeding behavior, and changes in the fish's metabolism.

**Table 2:** Effect of silver nanoparticles on fish growth and water quality.

	Fish	Size of AgNP	Size of fish (Weight of fish)	Treatment of AgNP	Effect on growth	Water quality	Results	References
AgNP	Nile tilapia *(Oreochromis niloticus)*	80–90 nm	Fingerlings (W = 10.32 ± 1.10 g) (L = 6.10 ± 0.5 cm)	(0, 10, 20, and 30μgL^-1^)	↑ AgNP exposure levels leads to ↓ in different growth indicators in a dose-dependent manner. The highest CAT, SOD, GPx, lysozyme, and respiratory burst activities were recorded with 10 μg AgNPs L^−1^. Enzyme activities ↓ with ↑AgNPs conc.	water temp 26 ± 1 ^◦^C; dissolved oxygen 6 ± 0.5 mg L^−1^; ammonia concentration 0.53 ± 0.07 mg L^−1^; and pH 7 ± 0.20.	The results revealed that growth performance of fish exposed to ↓ AgNPs conc improved significantly compared to othertreatments.	(Mabrouk et al., 2021)

Abalone viscera hydrolysates-AgNPs	Zebra fish *(Danio rerio)*	45–75 nm	–	0,6,9 and 18 μg/L)	6 and 9 μg/L ↑ growth of fish ↑ CAT, SOD and GSH in the liver and upregulated the expression of immune related genes	water temp of 25 ± 2 °C; dissolvedoxygen (DO) of 7.9 ± 0.1 mg. pH 7 ± 0.2.	AVH-AgNPs treatmentsof 6 and 9 μg/l promoted the growth and did not cause obvious damage to the gills, intestines, and livers of zebrafish.	(Ni et al., 2022)

AgNPs	(African catfish *Clarias garpiepinus)*	20 nm and 40 nm	L = 23.5–32 cm, W = 70–110	10 and 100 μg/L)	↑ in the concentration of liver enzymes proliferation of hepatocytes, infiltrations of inflammatory cells, pyknotic nuclei, cytoplasmic vacuolation, dilation in the blood vessel, hepatic necrosis, rupture of the wall of the central vein, and apoptotic cells in the liver of AgNPs-exposed fish	Water, temp, pH, and DO were measured daily (29.17 ± 0.27 °C, 8.5 ± 0.03, pH and 34.47 ± 11.99 mg/L DO)	Changes in color and loss of appetite were also recorded for some fish, especially in those fish exposed only to silver 20 nm/100 μg/L AgNPs.	(Naguib et al., 2020

AgNPs	Common carp (*Cyprinus carpio*)	58 ± 11 nm	L: 4.61 ± 0.88 cm, body weight: 2.56 ± 1.66 g)	AgNPs [0, 0.25 mg/L, 0.50 mg/L, 0.75 mg/L (1/2 LC50), 1.00 mg/L, and 1.25 mg/L] AgNO3 at a conc of 0.05 mg/L (1/2 LC50)	↑ GST, MDA, MT, and metallo thiondildehyde ↓ in fish gill membrane fluidity, ↑ the level of lipid peroxidation, and inhibited Na^+^ /K^+^ ATPase enzyme activity	The dissolved oxygen, pH, and temperature of the experimental water thus obtained were 4.5–6.0 mg/L, 7.13 ± 0.31, and 18 °C ± 2 °C,	The mechanism of AgNPs membrane toxicity involves the oxidization of long-chain omega-3 unsaturated FAs to saturated FAs via lipid peroxidation, resulting in ↓ membrane fluidity and ultimately the destruction of the normal physiological function of the fish gill membrane.	(Xiang et al., 2020)

AgNPs	Common Molly (*Poecilia sphenops*)	___	W = 5 ± 0.5 g	0, 5, 15, 25, 35, 45 and 60 mg L^−1^	Submitting a fish to higher concentration than 10 mg L^−1^ has adverse effects on reproductive system and blood parameters. Total protein, albumin, cholesterol and triglycerides ↑ in fish submitted to Ag-NPs (concentrations of 5–15).	temperature 27 ± 1 °C, pH 7–7.8, NH_3_ < 0.02 mg L^−1^, BOD 850 ± 50 mg L^−1^,total hardness 200 mg CaCO_3_ and NaCl < 1 mg L^−1^.	The results show a positive correlation between mortality rate and Ag-NP concentration. RBC, WBC and hematocrit were significantly decreased in fish exposed to Ag-NPs	(Vali et al., 2022)
	Rainbow trout *(Oncorhynchus mykiss)*	6–16 nm	L:23.75 ± 1.15 mm; body weight 0.012 ± 0.002 g)	0.1 mg/l. 0.2 mg/l, and 0.4 mg/l	Growth of the rainbow trout was decreased. ↑ AgNP exposure levels leads to ↓MCV and MHC, ↑ MCHC as the conc of AgNPs inc. Conc of liver enzymes ↑ in all the treatment groups than those of control. TP and AL conc ↓ as the conc of AgNPs ↑. AgNP exposure levels leads to ↓	Temp:15 °C, pH = 7.2–7.8, and DO = 6 mg/l	Silver nanoparticles induce significant changes in haematological parameters of rainbow trout	(Imani et al., 2015)
	Rainbow trout *(Oncorhynchus mykiss)*	Less than 100 nm	23.57 ± 1.15 nm body length and 0.12 ± 0.02 g body mass	1.5 mg l^−1^	Survival and growth of rainbow trout in the control and in the groups exposed for 28 days to silver (1.5 mg/L) was decreased	Temp: 18.25 ± 0.77 °C, pH 8.4 ± 0.18, and the concentration of dissolved oxygen was 8.70 ± 0.25 mg/L	Survival of fish from the control group was higher compared to the fish intoxicated with AgNPs	(Ostaszewska et al., 2018)

Low concentrations exposure of silver nanoparticles (10 µg/l) improved the growth performance of common carp. The study showed that the fish exposed to silver nanoparticles had significantly higher body weight, length, and condition factor in comparison to the control group (Mabrouk et al., 2021). The researchers suggested that the silver nanoparticles may have acted as a dietary supplement and enhanced the fish's growth (Pirani et al., 2021).

### Impact of silver nanoparticles on fish health

Silver and other nanoparticles have drawn a lot of attention in the aquaculture industry as a specific tool for managing numerous fungal, bacterial and viral infections, particularly with the growing problem of antibiotic-resistant microorganisms. Aeromonas species, the most frequent harmful bacterial strain endangering the aquaculture sector, were found to have bactericidal action against silver nanoparticles (Mahanty et al., 2013). Short-term exposure of *Oncorhynchus mykiss* challenged with *A. salmonicida* to silver nanoparticles (100 g/Lh^−1^) resulted in no mortality or clinical signs as compared to controls (challenged but not exposed to AgNPs) (Shaalan et al., 2018). In many aquatic animals, AgNPs has shown antibacterial efficacy against *A. hydrophila* and *Vibrio harveyi* (Adeniji, Nontongana, Okoh, & Okoh, 2022). Furthermore, appropriate dietary amounts of silver nanoparticles could increase aquatic organism survival, zootechnical efficiency, and metabolic rank, as well as decrease stress from both abiotic and biotic sources (Kumar & Rajeshkumar, 2018).

On the other hand, because of their widespread use in various industries, AgNPs pose a rising threat to aquatic ecosystems (Islam, Jacob, & Antunes, 2021). The release of silver nanoparticles into natural water bodies could occur through the manufacturing or disposal of nanoparticle-containing products. Depending on the time of exposure, concentration, and size of the studied AgNPs, fish exposed to greater levels of silver nanoparticles showed varying degrees of reactions, including inflammatory conditions, immunological oppression, stress to metabolism, biochemistry disruption, and growth retardation (Mahanty et al., 2013). As a result, even at low exposure levels due to contamination of water or during illness treatment, a full evaluation of silver nanoparticles toxicity could be conducted.

### Use of silver nanoparticles in controlling fish disease

Nanoparticles have a wide range of applications in aquaculture. There is limited knowledge regarding the impact on aquatic organisms. The use of silver nanoparticles in water for treating fungal diseases has been shown to be harmful to young trout, however a silver nanoparticle-coated water filter that is used in fish farming could prevent rainbow trout from contracting fungal infections. Fish health can be improved by using nanotechnological applications in the aquaculture sector, nanoscale delivery of veterinary goods in fish food via permeable nanostructures, and nano sensors for identifying infections in fish farming systems. Consequently, nanoparticles have demonstrated immense promise in a number of pond-ecosystem scenarios. Dealing with infectious diseases produced by pathogenic bacteria was one of the most difficult challenges in aquaculture. As an outcome of the widespread application of antibiotics in fish aquaculture, several pathogenic microorganisms in fish have acquired resistance to the commonly used antibiotics. As a result, fresh treatment approaches for addressing this issue are required. The use of silver nanoparticles as substitute antimicrobial agents in aquaculture to counteract the emergence of microbial resistance to antibiotics has been proposed as an alternative strategy to the problem of antimicrobial resistance (McNeilly, Mann, Hamidian, & Gunawan, 2021). A number of metal nanoparticles have demonstrated potential antibacterial effects against fungal, bacterial, and viral diseases through a variety of mechanisms such as cell membrane/cell wall disintegration, disruption of protein transports, inactivation of key enzymes and others (Nasr-Eldahan et al., 2021).

One of the biggest challenges to the aquaculture industry's ability to operate sustainably is fish sickness, which annually costs millions of dollars in lost revenue. Because the bacteria are so common in aquatic environments, there are many opportunities for animals, mostly fish and amphibians, to come into contact with and consume organisms. Due to the rapid spread of antibiotics through water, the use of antimicrobial medications in aquatic medicine has the potential to seriously harm the ecosystem (Szymańska et al., 2019). The antibacterial properties of metallic silver have been known for a very long time, and a range of silver compounds, including as silver dressings, silver nitrate, silver zeolite, and silver nanoparticles, are still utilised today. New antibacterial drugs are necessary due to the rise in bacterial illness outbreaks in the aquaculture sector and the emergence of bacterial resistance. Using nanoparticles as antimicrobial medications is one option. One of the most efficient metallic nanoparticles is silver, which has shown to be effective against certain bacteria, viruses, and other eukaryotic microorganisms.

Sarkar et al. (2012) studied the antibacterial activity of silver nanoparticles from Cedrus deodar leaf extracts against the principal fish pathogen *Aeromonas hydrophila* in order to improve fish health management. Another study by Kandasamy, Alikunhi, Manickaswami, Nabikhan, & Ayyavu, 2013, investigated the effect of leaf extract from the coastal plant *Prosopis chilensis* on the synthesis of silver nanoparticles using AgNO3 as a substrate to determine the antibacterial potential of silver nanoparticles against pathogenic vibrio species in the prawn *Penaeus monodon*. The silver nanoparticles were found to be capable of suppressing the vibrio pathogens *Vibrio cholerae*, *Vibrio harveyi*, and *Vibrio parahaemolyticus* in a disc diffusion experiment. This antimicrobial action outperformed that of the leaf extract. Silver nanoparticle-fed *Penaeus monodon* prawns had higher survival rates, which were linked to immunomodulation as seen by increased haemoglobin levels, phenol oxidase activity, and antibacterial *P. monodon* haemolymph levels comparable to control levels.

### Silver nanoparticles to improve water quality in aquaculture

The aquaculture requires more water than other agricultural techniques, and because water is becoming increasingly scarce globally, the intense intervention that aquaculture practises have developed has a direct impact on the environment. Moreover, not used fish food, excretion products, faeces, chemicals, and antibiotics produced more waste during organism formation and released it into the environment near these production farming facilities (Plascencia & Almada, 2012). The process of regularly replacing the water in ponds with freshwater was for a long time the one employed most often to improve the quality of the water. Yet, modest to medium-sized aquaculture systems may require several hundred cubic metres of water every day. According to Khan, Naushad, Al-Gheethi, & Iqbal, (2021), nanotechnology can be used to create membranes that are developed with nanoparticle compounds to remove trash and organic matter from water while also disinfecting it with silver bactericide nanoparticles.

Pradeep (2009) reviewed the use of nanoparticles in water disinfection to prevent the existence of bacterial and viral pathogens. However, this study shows a cost-benefit analysis is necessary, because the technology can already be expensive. Fewtrell (2014) showed how bioaccumulation of nanoparticles in water could impact fish culture. Toxicology research is required to decide whether to utilise them in aquaculture. This condition was replicated by another study in which AgNPs concentrations were measured in rainbow trout at three different life stages (alevin, fry, and fingerlings) at 100, 32, 10, 3.2, 1, 0.32, 0.1, and 0.032 mg/L. CL50 to 96-hour estimates were 0.25, 0.71, and 2.16 mg/L, respectively. These values demonstrate a greater sensitivity to early life stages. Moreover, a decrease in chloride and potassium levels as a function of dose concentration and an increase in cortisol and cholinesterase in juvenile stages were seen in blood plasma. This simply serves to demonstrate that silver nanoparticles used as a disinfectant in the aquaculture business or as direct applications to aquatic habitats in species raised for human consumption cannot be tolerated. Some other studies related to the use of silver nanoparticles in controlling water quality are presented in Table 2.

The ovules that had been fertilised were then moved to hatching systems, where they now receive water from cover filters that had nanoparticles in them. Using survival rates from fertilisation to the last stage of vitellus sac absorption, the filter's effectiveness was assessed. In comparison to the control test, the results showed that filters containing silver nanoparticles at 5 % increased survival rates by 4.56 % from fertilisation to the swimming phase. Significant differences were not evident in these findings. In almost all cases, adding more activated carbon to filters together with half-coated silver nanoparticles causes an increase of 11.24 % in alevin stage survival rates. In tests using filters covered in silver nanoparticles, infections were not seen during the incubation period. As a result, the final results showed that using silver nanoparticles directly in filters was very efficient in preventing fungus infections in rainbow trout semi-recirculating systems, without noting damages when directly inoculation was used. Sharma and Jha (2017) on the other hand, claimed that some nanoparticle bio-filters had been designed to get rid of ammonia, a chemical component that, in high water concentrations, is toxic to many aquatic creatures. This study makes it possible to confirm that nanoscale powder, which is ultrafine in nature, may also be utilised to remove contaminants from water. These granules are a useful tool for decomposing organic molecules into more straightforward and harmless ones.

**Fig. 3:**
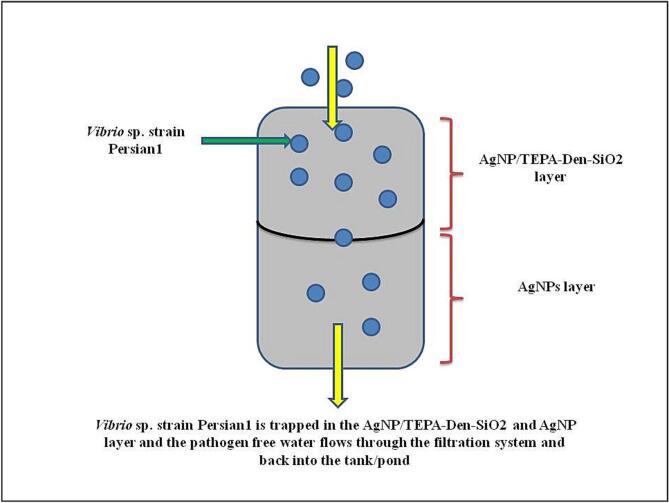
Water filtration system employing AgNPs with a silver absorbent layer (Adapted from De Silva et al., 2021).

Carbon and cyclodextrin nanotubes were produced and coated with silver nanoparticles in a different study by Lukhele, Mamba, Momba, & Krause (2010) to filter water samples where *E. coli* and *V. Cholearae* were found. These materials were tested using nanotubes, and the final experiments result showed a reduction in colony forming units (CFU). According to Sarkheil, Sourinejad, Mirbakhsh, Kordestani, and Johari (2016), a simplified diagram (Fig. 3) of the application of silver nanoparticles in a water filter system with a silver absorbent layer against the Vibrio sp. strain Persian1 infecting Pacific white prawns is reported. There were two filter systems developed: one with silver absorbent and one without. The results show that when compared to the filter system without the silver absorbent after 2, 6, and 12 h, the filter system with the silver absorbent demonstrated much greater bacterial clearance.

### Use of AgNPs in fish packaging

Fresh fish is highly perishable, thus any packaging that might lengthen its shelf life would be beneficial. This worry has long existed. This can be done in a number of different ways. Firstly, there are nano polymers and coatings available for packaging strengthening, which may lessen the likelihood of bruising or mechanical damage to packaged fish fillets. When compared to some conventional plastics, nano packaging is anticipated to be biodegradable since it can be manufactured from natural nanoscale polymers like cellulose and starch or chitosan particles. Moreover, strong and lightweight nano packaging for the meat industry has been proposed. A promising type of active food packaging that extends food shelf life and lowers the danger of infections is AgNPs-based antimicrobial packaging (Carbone, Donia, Sabbatella, & Antiochia, 2016). Now utilised in fish and fish product packaging, nano-materials are added to metals and metal oxides like silver, gold, zinc oxide, silica, titanium dioxide, alumina, and iron oxides, among others.

### Prevention of bio-filing

Biofouling refers to the accumulation of unwanted microorganisms, such as bacteria, algae, and marine organisms, on surfaces submerged in water. This can occur in various industries, including marine, biomedical, and water treatment. Silver nanoparticles can help prevent biofouling in aquaculture. It can be incorporated into coatings applied to aquaculture infrastructure, such as nets, cages, and pipes. These coatings can release silver ions, which exhibit antimicrobial properties, inhibiting the attachment and growth of fouling organisms. The nanoparticles can be mixed with appropriate binders to ensure adhesion to the surface and controlled release of silver ions. Silver nanoparticles can be incorporated into coatings or paints that are applied to surfaces vulnerable to biofouling. These coatings release silver ions, providing continuous protection against fouling organisms. The nanoparticles can be mixed with polymer matrices or binders to ensure their adhesion to the surface. Moreover, silver ions are highly toxic to microorganisms, and even low concentrations can have a significant antimicrobial effect. The release of silver ions can occur gradually over time, providing long-term protection against biofouling (Moerman, 2014).

### Nano-biosensors

Nanotechnology based biosensors can be used by the aquaculture industry to regulate microbial populations (Moges, Patel, Parashar, & Das, 2020). The National Aeronautics and Space Administration have developed a biosensor based on sensitised carbon nanotubes that can detect tiny levels of germs in food and water, including bacteria, viruses and parasites. Nano colloidal sliver is a beneficial by-product of nanotechnology that acts as a catalyst. It acts by making an enzyme necessary for their metabolism inactive on a wide range of parasites, bacteria, fungi, and viruses. Contrary to bacterium strains resistant to antibiotics, colloidal sliver is not known to cause the development of such strains. Even *Staphylococcus aureus* that is resistant to methicillin can be killed by silver nanoparticles. Tracking nano sensors, like “Smart fish,” are being created. These fish may be outfitted with sensors and locators that broadcast data about the fish's position and health to a central computer. This type of technology could be used to manage fish or cognitive cage systems.

### Toxicity of silver nanoparticles in fish

When studying the toxicity and interactions of AgNPs with biota, they are typically compared to either larger (micron sized) Ag particles, similar sized particles made of other materials, or dissolved silver ions (McGillicuddy et al., 2017). Ion toxicity in freshwater fish species has been widely studied in vivo, with certain freshwater fish species having LC10 values as low as 0.8 g/L. In solution, Ag ions can enter the gills via the Na^+^ channel coupled to the proton ATPase in the apical membrane, travel to the apical membrane, and block the Na + Potassium ions ATPase, impacting the ion regulation of Na^+^Cl^-^ ions across the gills. There are significant physiological repercussions at high concentrations (M), such as blood acidity, which can eventually lead to circulatory collapse and mortality. Only a few studies have looked at how AgNPs affect fish in vivo (Scown et al., 2010). Early evidence suggests that 10–80 nm AgNPs have an impact on early life development, including nervous system defects, irregular heartbeats, and mortality (Asharani, Wu, Gong, & Valiyaveettil, 2008). Silver nanoparticles can also accumulate in the gills and liver tissue, limiting the fish's ability to cope with low oxygen levels and causing cell damage (Scown et al., 2010).

**Fig. 4:**
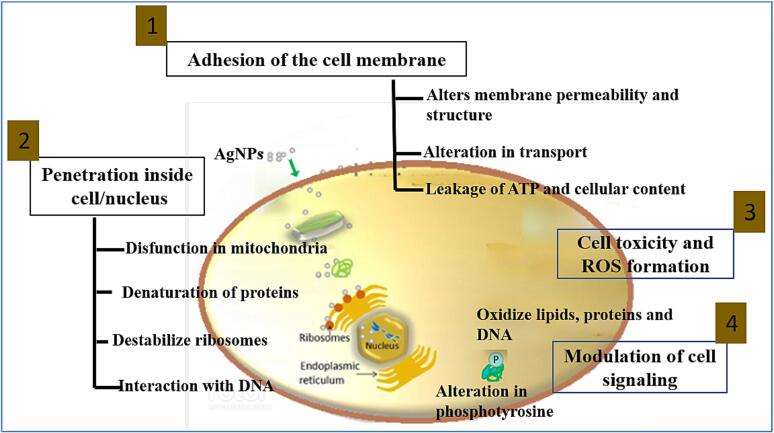
Silver nanoparticle accumulation into the cell membrane.

Moreover, even for the same species, the limit at which such changes happen varies across experiments. Such variance may reveal changes in experimental conditions chosen and/or unspecified distinctions in particle actions or character. In general, Japanese medaka and juvenile zebrafish are known to be more sensitive to AgNPs than to equal mass concentration levels of AgNO_3_, at least under conditions that achieve maximum free ion Ag concentrations for the later (Wang, & Wang, 2014) According to some authors, particulate solubilization is unlikely to be the cause of the toxicity (Chae et al., 2009). Other experiments with similar masses of AgNPs and AgNO3 (as low as 10–20 ng/L for embryonic studies and 1 mg/L for juveniles) have evidenced the contention for distinct action mechanisms, with relative uptake and gene expression responses for AgNPs compared to AgNO_3_ (Asharani, Wu, Gong, & Valiyaveettil, 2008). Similarly, zebrafish and rainbow trout have distinct absorption of Ag substances. In zebrafish embryos, high exposure concentrations (0.4 and 100 mg/L) indicate that AgNPs aggregation were assimilated into blood vessels, yolk, brain, skin, and heart, whereas Ag ions are only found in organelles, nucleus, and the yolk. Moreover, the mechanism explaining how these silver nanoparticles accumulate the cell membrane is depicting in Fig. 4.

## Challenges with respect to the use of AgNPs in aquaculture

There are several problems with the usage of AgNPs in aquaculture that need to be resolved. The potential environmental impact of using AgNPs is one of the most important concerns (Ihtisham et al., 2021). Research is required to find out about their persistence, bioaccumulation, potential toxicity to creatures other than targets, and capacity to disrupt aquatic ecosystems. AgNPs have a reputation for being easily aggregating and dissolving, which can alter their properties and reduce their efficacy (Bélteky et al., 2019). To maximize their effectiveness, AgNP stability in aquaculture systems is a problem that needs to be resolved. Furthermore, the development of safe and efficient AgNP delivery devices is essential. To ensure the regulated and targeted release of AgNPs while minimising any potential detrimental effects, encapsulation techniques and controlled-release formulations must be investigated.

## Conclusion

Nanotechnology is a developing field with numerous uses in aquaculture including the treatment of pathogenic infections, water quality management, monitoring fish health etc. which ultimately helps in the sustainable production etc. In recent years, there has been a lot of discussion about the role of silver nanoparticles (AgNPs) in aquaculture. AgNPs have unique features, such as antibacterial activity that make them potential candidates for use in aquaculture systems. Besides, AgNPs represent a remarkable advancement in the field of food science and technology with specific relevance to the aquaculture sector. Their synthesis through eco-friendly methods, such as plant extracts or microorganisms not only align with sustainability goals but also yield nanoparticles with unique properties, including antimicrobial and antioxidant capabilities. The use of plants in the production of green nanoparticles is a fascinating and emerging topic of nanotechnology that has a substantial impact on the environment while also contributing to nanoscience's long-term sustainability and growth. These green plant-based nanoparticles may find applications in catalysis, medicine, agriculture, food packaging, water treatment, dye degradation, antibacterial, antifungal, aquaculture, sensors, imaging, biotechnology, and other biological fields. Apart from that, extensive studies have been undertaken to investigate the potential of AgNPs in increasing the growth of aquatic organisms, health and other uses as well. Several studies have shown that AgNPs can effectively limit the growth of numerous pathogens in aquaculture system, including bacteria, fungus and viruses. Their antibacterial qualities aid in the prevention of diseases and the survival of farmed organisms. Furthermore, AgNPs have the potential to be used as an alternative to traditional antibiotics, which can help to reduce antibiotic use in aquaculture and reduce the risk of antimicrobial resistance.

## Future prospective

The role of silver nanoparticles in aquaculture offers a lot of promise. While there has been substantial progress in understanding their antibacterial characteristics and potential benefits, more research is required to address concerns regarding their environmental impact, formulation, nanoparticle size, animal health and regulatory factors. By addressing these issues, AgNPs can contribute to long-term aquaculture practises, improve disease management and improve the overall performance of aquaculture systems. However, there is a considerable gap in the knowledge of long-term consequences of AgNPs on aquatic ecosystems. The persistence, bioaccumulation and potential toxicity to non-target organisms should be studied. Further research should also be conducted to investigate the mechanisms of action of AgNPs in boosting immune responses in aquatic organisms. This can aid in the optimisation of its application and the identification of any risks associated with prolonged exposure.

## Declaration of competing interest

The authors declare that they have no known competing financial interests or personal relationships that could have appeared to influence the work reported in this paper.

## Data Availability

Data will be made available on request.
